# Active and Passive Maternal Smoking During Pregnancy and Birth Outcomes: A Study From a Developing Country

**DOI:** 10.5334/aogh.3384

**Published:** 2021-12-03

**Authors:** Shereen Hamadneh, Jehan Hamadneh

**Affiliations:** 1 Department of Maternal and Child Health, Faculty of Nursing, Al al-Bayt University, Mafraq, Jordan; 2 Department of Obstetrics and Gynaecology, Faculty of Medicine, Jordan University of Science and Technology, Irbid, Jordan

## Abstract

**Background:**

Smoking is one of the modifiable risk factors for adverse maternal and neonatal outcomes and is associated with low birth weight, preterm birth, respiratory, antepartum and intrapartum stillbirth, and perinatal death as well as long-term morbidity in offspring and sudden unexpected infant death. The rate of smoking in low- and middle-income countries is still relevantly high, and Jordan is no exception.

**Objective:**

To investigate the effect of active and passive smoking during pregnancy on adverse pregnancy outcomes.

**Methods:**

The case-control study was conducted in Jordan in June 2020. Healthy women with full-term singleton pregnancy (n = 180) were interviewed and stratified into three groups: Group I, active smokers; Group II, passive smokers; and Group III, nonsmokers. The study variables included demographic data, current pregnancy history, cotinine level of mothers and newborns, and perinatal outcomes. Statistical analysis was performed using the application package IBM SPSS 25. Various algorithms of statistical analysis were used depending on the type of distribution of feature and data quality. The threshold for statistical significance was set at *p* < 0.05.

**Results:**

Active smokers had significantly lower gestational age at delivery compared to passive and nonsmoking women (*p* = 0.038 and *p* = 0.003, respectively). Neonates from active smoking mothers had significantly lower birth weight compared to neonates from passive and nonsmoking women (*p* = 0.016 and *p* = 0.019, respectively), significantly lower head and chest circumferences compared to babies from passive smokers (*p* < 0.001 and *p* = 0.036, respectively), and significantly lower first-minute Apgar score compared to those from nonsmoking women (*p* = 0.023). The urine cotinine level was significantly higher in both active and passive smoking women (both *p* < 0.01), and it was significantly higher in newborns who had been exposed to smoking in utero despite maternal active or passive smoking status (both *p* < 0.001). There was a weak negative correlation between urine cotinine level and birth weight: *r* = –0.14 for maternal cotinine level and *r* = –0.15 for neonate cotinine level.

**Conclusions:**

The current study illustrated that smoking during pregnancy leads to offspring with reduced birth weight, birth length, and head and chest circumference; reduces delivery gestational age; and lowers the first-minute Apgar score. Our study findings highlight the need for further research issued to smoking effects on perinatal outcomes, the implementation of actions to develop cessation interventions in the preconception period, and an evaluation of useful interventions to enhance a smoking-free environment during pregnancy.

## Introduction

Smoking is a modifiable risk factor for adverse maternal and neonatal outcomes and is associated with maternal, fetal, and infant morbidity and mortality [7].

As shown in previous research, active and passive maternal smoking during pregnancy increases the risk of having a child with low birth weight [27,34] and significantly increases other negative pregnancy outcomes, such as preterm birth [17,21], respiratory distress [1], antepartum and intrapartum stillbirth [5], perinatal death [24], long-term morbidity in offspring [32], and sudden unexpected infant death [3].

Birth weight, length, and head and chest circumference at birth are the main indicators of fetal growth suppressed by maternal smoking [15]. It is not clear from the current body of evidence whether maternal smoking specifically affects head growth, though maternal smoking during pregnancy negatively affects fetal brain development [16].

The Apgar score is used as a standardized index of newborn health at birth and is strongly associated with the risk of neonatal and infant death [4]. According to [35], babies of smoking mothers had lower Apgar scores compared to those of nonsmokers. However, smoking during pregnancy is not an independent predictor of the Apgar score. It remains unclear whether quitting smoking during pregnancy affects the Apgar score [35].

The rate of smoking in low- and middle-income countries is still relevantly high [22,23,29]. The prevalence of active and passive smoking in Jordan is high [9,10,11,13], and it mirrors the husbands’ active smoking patterns [11,29]. Paternal smoking is considered an independent risk factor for fetal growth restriction [6] and stillbirth despite the maternal smoking status [28].

This study aimed to investigate the effects of active and passive smoking during pregnancy on adverse pregnancy outcomes.

## Methods

The case-control study was conducted at the main hospital in Jordan (King Abdullah University Hospital (KAUH)) using a semistructured questionnaire in June 2020.

Inclusion criteria for the study were as follows: full-term singleton pregnancy, absence of chronic diseases (e.g., cardiovascular, kidney, endocrine diseases) and pregnancy complications (gestational hypertension and diabetes mellitus), accommodation at the north of Jordan, prenatal car, and delivery and postnatal care at KAUH Obstetrics and Gynecology Department as well as an agreement to participate in research and completion of at least 75% of the questionnaire.

The Institutional Research Board (IRB) at Jordan University of Science and Technology (University Review Committee for Research on Human) approved all study activities. All participants in this research were voluntary, and anyone could stop participating in the interview at any time. The consent form was prepared to get the agreement of the participants to be interviewed, and only those who agreed to participate in this study were interviewed. The consent form excluded the possibility of unjustified deception, undue influence, and intimidation. The agreement was signed only after prospective subjects were adequately informed. Their decision on whether to participate did affect the doctor-patient relationship or any other benefits to which they are entitled. Personal information about subjects will never be disclosed, and the data collected will remain confidential.

All women included in the study (n = 180) were interviewed according to the questionnaire designed and developed by our team and included yes/no questions, select from a list questions, and short answer questions in the Arabic language. All participants were stratified into 3 groups according to their smoking status: Group I, current active smokers; Group II, women with current exposure to secondhand smoke; and Group III, nonsmoking women, neither active nor passive.

The study variables included demographic information (maternal age, education, work status, environment status, smoking behavior and attitudes), current pregnancy history (parity, mode of delivery), and perinatal outcomes (birth weight, length, head and chest circumferences, Apgar score at 1 and 5 minutes, NICU admission).

Data for this study came from a face-to-face survey with pregnant women and laboratory reports. Data was entered into a unified computer database and analyzed by our team.

Statistical analysis was performed using the application package IBM SPSS 25 (SPSS: An IBM Company, New York, USA). The character of data distribution was evaluated with the W-criterion of Shapiro-Wilk. Various algorithms of statistical analysis were used depending on the type of distribution of feature. Absolute and relative indicators (%) were used to represent the qualitative characteristics. Quantitative data were presented by central tendency and dispersion: the mean value (M) with a 95% confidence interval (CI). A comparison of three independent groups on one or several signs, having at least one of the groups of distribution different from the normal or if the type of distribution was not analyzed, was carried out by checking the statistical hypotheses about the equality of middle rank using the Mann-Whitney U-test. Analysis of contingency tables (χ2) was used to assess the differences in relative values. Fisher exact test (*p*) was applied at frequencies less than 5. The threshold for statistical significance was set at *p* < 0.05.

## Results

### Brief Characteristic of the Studied Group

All women who participated in the study were married. The average maternal age was 30.23 ± 1.47, 28.85 ± 1.50, and 31.1 ± 1.34 years among active smokers, passive smokers and nonsmokers, respectively. It was significantly lower in the group of passive women smokers compared with women who do not smoke (*p* = 0.027). Active smokers were significantly less educated than passive smokers and nonsmokers (*p* < 0.001), and most of them did not work compared to nonsmokers (*p* = 0.037). Additionally, their family monthly income was significantly lower than in the group of passive smokers (*p* = 0.006). Approximately 13% of active smoking women were nulliparous, while 43% of passive smokers were nulliparous women (*p* < 0.001). Both active and passive smoking groups were less likely to follow up with perinatal care clinics compared to women who did not smoke (*p* = 0.029). Brief characteristics of the studied groups are represented in Appendices Table 1.

### Smoking Behavior and Attitudes in Studied Groups

Most smoking women (both active and passive) reported their husband is also a smoker and smokes at home (63% [50.68–74.38] and 80% [68.22–88.17], respectively), while only 2% (0.30–8.86) of nonsmoking women declared about a smoking husband (*p* < 0.001).

We found that most women started to smoke at the age of 20, and more (43% [31.57–55.89], 33% [22.73–45.94]) classified themselves as moderate and heavy smokers. All of them smoke at home, and 20% (11.83–31.78) smoke at the workplace.

Our study revealed that many smoking women received information about the hazardous effects of smoking during perinatal visits (43% [31.57–55.89]), and 83% (71.96–90.68) were aware of the effects of smoking on perinatal outcomes. However, only 13% [6.91–24.16] of women quit smoking cigarettes before or in the early termination of pregnancy. Approximately 23% (14.44–35.43) decreased the frequency of smoking during their pregnancy, 13% (6.91–24.16) continued to smoke with the same frequency as before, and 23% (14.44–35.43) of women tried to stop their habit, but without success.

### Pregnancy Outcomes

Active smoking women had significantly lower gestational age at delivery compared to passive smoking and nonsmoking women (*p* = 0.038 and *p* = 0.003, respectively). There were no differences in the rate of cesarean section between study groups (*p* > 0.05).

Neonates from active smoking mothers had significantly lower weight at birth compared to neonates from passive smoking and nonsmoking women (*p* = 0.016 and *p* = 0.019, respectively) as well as significantly lower head and chest circumferences compared to babies from passive smoking mothers (*p* < 0.001 and *p* = 0.036, respectively). Additionally, we found that neonates from active smoking women had a significantly lower 1-minute Apgar score compared to those from nonsmoking women (*p* = 0.023), while there were no differences in the 5th-minute Apgar score. The rate of NICU admission did not differ among the 3 groups.

The main pregnancy outcomes are represented in Appendices Table 2.

### Urine Cotinine Level in Mothers and Newborns

The urine cotinine level was significantly higher in both active and passive smoking women compared to nonsmoking women—43.27 ± 15.57 ng/mL, 1.08 ± 0.68 ng/mL, and 0.17 ± 0.05 ng/mL, respectively (both *p* < 0.01). Additionally, the urine cotinine level was significantly higher in newborns who had been exposed in utero (despite maternal active or passive smoking status)—42.53 ± 15.81 ng/mL, 0.42 ± 0.14 ng/mL, 0.15 ± 0.04 ng/mL for the active, passive, and nonsmoking group, respectively (both *p* < 0.001). A Pearson correlation test revealed there was a weak negative correlation between urine cotinine level and birth weight—*r* = –0.14 for maternal cotinine level and *r* = –0.15 for neonate cotinine level.

## Discussion

Smoking is considered one of the preventable risk factors for poor perinatal outcomes. Over the past several decades, many studies related to this issue have been conducted worldwide. Moreover, most of them have shown that smoking is a serious threat to public health, and this problem needs to be addressed at the level of the health care system [6,7].

This study, aimed at identifying the effects of smoking on perinatal outcomes, underscores the importance of smoking cessation interventions, especially in the context of high smoking rates among men and women of childbearing age [10,11,13].

Our study revealed that active smoking women were less educated and had less monthly family income compared to passive smoking and nonsmoking women. These findings replicate those of a similar study, where pregnant women with less than a high school education were more likely to smoke as compared to women with bachelor’s degree or higher [19].

The current study showed that active smoking women were likely to deliver an earlier gestational age compared to passive smoking and nonsmoking women. This finding is also suggested by several studies on the risk of preterm birth in smoking mothers [17,21]; however, we cannot equate these studies, since, in our study, the inclusion criterion was a full-term pregnancy.

According to Li and colleagues (2019), smoking women were more likely to have a caesarean section for nonreassuring fetal status [20]. However, in our study, there were no differences in the mode of delivery between women despite their smoking status.

We found a significantly lower 1-minute Apgar score in newborns from active smoking mothers compared to those from nonsmoking women, while the 5-minute Apgar score did not differ by maternal smoking status. The rate of NICU admission was similar between the study groups. Similar results were shown by Kharkova and colleagues (2017) [18], though another study found a significant increase in NICU admission in the smoking cohort [20]. Thus, we cannot accept smoking as the only factor that affects the Apgar score and the need for NICU admission [35].

Our study revealed the birth weights of newborns from active smoking mothers were significantly less than those from passive smoking and nonsmoking women, and secondhand exposure did not influence birth weight. Additionally, we found a tendency for smaller length and head and chest circumferences in newborns from active smoking women compared to those from nonsmoking mothers. These findings are strongly supported by similar studies, where maternal active smoking was associated with a lower mean birth weight [14,25], smaller length and head circumference [1,2,33], and abnormal body proportions [18,30].

We found that urinary cotinine levels in women and newborns were negatively associated with birth weight. However, this association was very weak. The only study that assessed the relationship between cotinine levels and anthropometric data in newborns found that the level of urine cotinine in newborns had a strong negative association with birth weight [8].

Furthermore, there is a high rate of smoking among Jordanian pregnant women, approximately 18% [9], and many were exposed to tobacco smoking by their husbands [9]. The smoking rate in Jordan is among the highest rates in the world, with a high exposure of smoking indoors and a lack of policy restrictions in developing countries [9,11,12,13,31]. This is a leading factor for stillbirths and sudden deaths among infants, which need more intervention programs [9,11,13,29]. Moreover, comprehensive smoke-free policies must be ensured in developing countries. In particular, for some health outcomes that are strongly influenced by active and passive smoking, keep in mind that smoking is a common habit and rates are higher among developing country communities [9,11,13,26].

### Strengths and Limitations

The small sample size and the fact that the study sample excluded high-risk pregnant women and women with preterm birth means our results cannot be extrapolated to all pregnant women in general or other regions of Jordan. These groups of women require additional study.

We discussed limited perinatal outcomes as per our exclusion criteria and did not take into account pregnancy complications such as preterm delivery, gestational diabetes mellitus, gestational hypertension, placental abruption, and fetal malformations, which can be caused by smoking. So, further studies should be directed to these issues too.

Our information on smoking behavior during pregnancy was based on self-reporting. Consequently, underreporting of maternal smoking may have occurred, resulting in potential for misclassification. However, to address this, we measured cotinine in mother-baby couples to assess the accuracy of self-reported data. This was a major strength of our study.

Take into consideration the necessary contribution to the literature on prenatal smoke exposure and risks on obstetric outcomes for women and children in Jordan. The main strength is the inclusion of laparotomy cotinine levels. Furthermore, this study was the first of its kind in Jordan and therefore provides necessary baseline information for further improvement on tobacco control research in the developing countries and the Middle Eastern regions.

## Conclusions and Recommendations

The current study illustrated that smoking during pregnancy leads to offspring with reduced birth weight, birth length, and head and chest circumference as well as reduced delivery gestational age and lower Apgar score. Moreover, we may conclude that argileh smoking (e.g., hookah) during pregnancy can also contribute to a reduction in newborns’ anthropometric measurements.

Our study findings highlight the need for continued study of the effects of smoking on perinatal outcomes and the need for targeted interventions to reduce and prevent tobacco smoking and tobacco smoke exposure during pregnancy. Further studies are needed to increase awareness of these adverse effects, to develop cessation interventions in the preconception period, and to evaluate useful interventions to enhance a smoking-free environment during pregnancy.

## Additional File

The additional file for this article can be found as follows:

10.5334/aogh.3384.s1 Appendices. Appendix Table 1 and Appendix Table 2.

## Author Information

Dr. Shereen Hamadneh:

1. Associate professor, Department of Maternal and Child Health, Princess Salma College of Nursing, Al-Bayt University, Mafraq, Jordan
2. A member of the Union of Jordanian Universities for Smoking and Tobacco Control
3. A scientific member of the executive council of the Center of Excellence for Scientific Research at the Scientific Society of Arab Nursing Faculties
4. A clinical nutritionist, a member of the Jordanian Diet Association
5. The chairman of the board of directors of the Happy Baby—Child and Family Association, Ministry of Social Development, Jordan
6. Director of Safe Child Care Program, Australian Aid Program, Jordan
7. A member of the board of directors of the Community Women Support Committee, Ramtha, Jordan
