# Supplementary material for: Active and Passive Maternal Smoking During Pregnancy and Birth Outcomes: A Study From a Developing Country
Source: Ann Glob Health. 2021 Dec 3;87(1):122. doi: 10.5334/aogh.3384 (PMC8641528; doi:10.5334/aogh.3384)
Supplement: Appendices. — Appendix Table 1 and Appendix Table 2. [file agh-87-1-3384-s1.pdf]

Appendices

Table 1. Characteristics of Studied groups

Characteristic	Group I, active smokers, n = 60 Absolute/ % [95%CI]	Comparison between Group I and control group Chi-square (*p*-value), □^{2} (*p*)	Group II, passive smokers, n = 60 Absolute/% [95%CI]	Comparison between Group II and control group Chi-square (*p*-value), □^{2} (*p*)	Group III (control), nonsmokers, n = 60 Absolute/% [95%CI]	Comparison between Group I and Group II Chi-square (*p*-value), □^{2} (*p*)
Maternal age, mean ± 95% CI	30.23 ± 1.47	$p = 0.386$	28.85 ± 1.50	$p = 0.027$	31.1 ± 1.34	$p = 0.189$
Marital status (married)	60/100 [93.98-100.0]	1.0*	60/100 [93.98-100.0]	1.0*	60/100 [93.98-100.0]	1.0*
Maternal education<br>- Secondary school<br>- College<br>- Bachelor's degree<br>- Postgraduate degree	28/46.67 [34.63-59.11]<br>4/6.67 [2.62-15.93]<br>26/43.33 [31.57-55.89]<br>2/3.33 [0.92-11.36]	< 0.001*	6/10 [4.66-20.15]<br>7/11.67 [5.77-22.18]<br>31/51.67 [39.31-63.83]<br>16/26.67 [17.14-39.01]	0.055*	2/3.33 [0.92-11.36]<br>2/3.33 [0.92-11.36]<br>44/73.33 [60.99-82.86]<br>12/20.0 [11.83-31.78]	< 0.001*
Working status (work)	18/30.0 [19.9-42.51]	0.037*	27/45 [33.09-57.51]	0.573*	32/53.33 [40.89-65.37]	0.244*
Family monthly income >500 JD	20/33.33 [22.73-45.94]	0.081*	32/53.33 [40.89-65.37]	0.398*	32/53.33 [40.89-65.37]	0.006*
Nulliparous	8/13.33 [6.91-24.16]	2.55 (0.110)	26/43.33 [31.57-55.89]	2.97 (0.085)	16/26.67 [17.14-39.01]	11.86 (<0.001)
Following up during pregnancy (more than 3 visits)	50/83.33 [71.96-90.68]	0.029*	50/83.33 [71.96-90.68]	0.029*	58/96.67 [88.64-99.08]	0.999*

* Fisher exact probability test

Table 2. Main Pregnancy Outcomes

Characteristic	Group I, active smokers, n = 60 Absolute/ % [95%CI]	Comparison between Group I and control group Chi-square (*p*-value), □^{2} (*p*)	Group II, passive smokers, n = 60 Absolute/ % [95%CI]	Comparison between Group II and control group Chi-square (*p*-value), □^{2} (*p*)	Group III (control), nonsmokers, n = 60 Absolute/% [95%CI]	Comparison between Group I and Group II Chi-square (*p*-value), □^{2} (*p*)
Gestational age at delivery, weeks	37.88 ± 0.58	*p* = 0.003	38.59 ± 0.36	*p* = 0.183	38.96 ± 0.40	*p* = 0.038
Mode of delivery (cesarean section)	32/53.33 [40.89-65.37]	0.83 (0.362)	34/56.67 [44.11-68.43]	1.63 (0.202)	26/43.33 [31.57-55.89]	0.03 (0.863)
Weight at birth, kg	3.0 ± 0.12	*p* = 0.019	3.21 ± 0.12	*p* = 0.905	3.2 ± 0.12	*p* = 0.016
Length at birth, cm	49.4 ± 0.64	*p* = 0.059	50.1 ± 0.54	*p* = 0.611	50.33 ± 0.74	*p* = 0.098
Head circumference, cm	34.27 ± 0.34	*p* = 0.094	35.06 ± 0.29	*p* = 0.265	34.75 ± 0.46	*p* <0.001
Chest circumference, cm	32.67 ± 0.46	*p* = 0.089	33.28 ± 0.36	*p* = 0.865	33.23 ± 0.48	*p* = 0.036
Apgar score in the 1st minute	7.63 ± 0.37	*p* = 0.023	7.97 ± 0.08	*p* = 0.059	8.07 ± 0.06	*p* = 0.083
Apgar score at the 5th minute	8.9 ± 0.21	*p* = 0.124	9.07 ± 0.09	*p* = 0.999	9.07 ± 0.06	*p* = 0.142
NICU admission	16/26.67 [17.14-39.01]	0.04 (0.842)	17/28.33 [18.5-40.76]	0.01 (0.999)	18/30.0 [19.9-42.51]	0.01 (0.999)
